# Genomics for natural product discovery: An annotated selection of World Wide Web sites relevant to the topics in *microbial biotechnology*

**DOI:** 10.1111/1751-7915.12353

**Published:** 2016-02-23

**Authors:** Lawrence P. Wackett

**Affiliations:** ^1^ Department of Biochemistry, Molecular Biology & BiophysicsBioTechnology InstituteUniversity of MinnesotaSt. PaulMN55108USA

## Natural Product Discovery Institute

### http://www.npdi-us.org

The Natural Product Discovery Institute contains a set of more than 30 000 fungal and actinomycete organisms that can be used for natural product discovery.

## Natural products library

### http://www.scripps.edu/shen/NPLI/thenaturalproductslibrary.html

The natural product library contains purified compounds, partially purified column fractions, and crude extracts from natural product‐producing organisms. The collection is available for screening.

## Natural product discovery based on genomics

### http://www.kelleher.northwestern.edu/publications?view=publication&task=show&id=221

This site describes a roadmap for analysing the genomes of Actinobacteria for natural product biosynthetic pathways.

## Phosphonic acid natural products via genome mining

### http://www.pnas.org/content/112/39/12175.full.pdf

In this study, data from the large‐scale sequencing of actinomycetes were mined to uncover new metabolic pathways for making phosphonic acid natural products. Biosynthetic phosphonic acids are often metabolite mimics that have interesting biological properties.

## Small molecules from human microbiota

### http://science.sciencemag.org/content/349/6246/1254766.abstract?rss=1

Microbes in the human gut are known to produce a large array of natural products and mining the genomes provides a pathway to new discovery and delineating differences between microbial populations in different people.

## Recent patents in genomics and natural products

### http://www.ncbi.nlm.nih.gov/pubmed/25185982

Recent papers relating to genomics and natural products are discussed in this article.

## Mass spectrometry and genomics: Natural products in cyanobacteria

### http://pubs.acs.org/doi/abs/10.1021/acs.jnatprod.5b00301

This paper deals with the discovery of a new class of di‐ and trichlorinated acyl amides with cannabinomimetic activity.

## Natural products for crop protection

### http://newsroom.dowagro.com/press-release/dow-agrosciences-radiant-genomics-announce-rd-collaboration-focused-natural-products-c

This announcement of an industrial cooperation illustrates the current interest in commercial genome mining for natural product discovery surrounding agricultural applications.

## Metagenomics for marine natural product discovery

### http://journal.frontiersin.org/article/10.3389/fmicb.2015.00890/full

This review article deals with the mining of metagenomic data for natural products that are generated by microbes from marine environments.

## Analysing secondary metabolism genes

### http://antismash.secondarymetabolites.org

This website provides a resource for submitting genome sequences to identify secondary‐metabolite biosynthetic gene clusters.

## Super Natural II

### http://bioinf-applied.charite.de/supernatural_new/index.php

This database contains the structures, properties and commercial sources for over 300,000 natural products.

## Universal natural product database

### http://pkuxxj.pku.edu.cn/UNPD/

This database contains structures and information on over 225,000 natural products to be used for virtual screening.

## iSMART database

### http://ismart.cmu.edu.tw

This multi‐functional database is composed of sub‐databases. One of those deals with small molecules important in Chinese medicine for virtual screening. Another database is valuable for docking and screening for drug development.

## iSNAP database

### http://www-novo.cs.uwaterloo.ca:8180/gnp/#!/isnap

This database allows for the identification of molecules based on LC‐MS/MS chromatograms.

## Genomics for natural product structure

### https://www.researchgate.net/publication/282812827_Using_Genomics_for_Natural_Product_Structure_Elucidation

This review details the value of genomic data for natural product identification.

## Microbial natural products

### https://nar.oxfordjournals.org/content/early/2015/10/05/nar.gkv1012.full

This paper describes PRISM, a computational resource for identifying natural product biosynthetic clusters, particularly polyketide clusters.

## Warp Drive Bio

### http://www.warpdrivebio.com

Warp Drive Bio formed to mine the genomes of actinomycetes for novel natural product clusters and identify a new generation of pharmaceuticals.

